# DESIGN AND EVALUATION OF AN ECOLOGICAL MOMENTARY ASSESSMENT TOOL FOR ASSESSING DEPRESSION IN DEMENTIA

**DOI:** 10.1093/geroni/igz038.1728

**Published:** 2019-11-08

**Authors:** Iulia Niculescu, Andrea Iaboni

**Affiliations:** University Health Network, Toronto, Ontario, Canada

## Abstract

Background. Assessing depression in people with dementia is challenging due to limitations of retrospectivity. Mobile Ecological Momentary Assessment (EMA) provides a novel approach in assessing depressive symptoms by collecting informant measures in intervals throughout the day, decreasing recall bias and increasing representativeness. Objective. The objective of this study is to design an EMA application for assessing depression in individuals with dementia. Methods. A literature review was conducted to determine commonly used and validated assessments for depression in dementia. Assessments were analyzed for common items, where items less commonly used (<50% of assessments sharing the item) or not relevant to be collected using EMA were excluded. Wording of items were also analyzed to develop the specific structure of questions for the EMA assessment. Results: Six assessments were found and demonstrated adequate performance outcomes. Items fell into either mood-related, dementia-related, vegetative, psychotic or positive mood symptom groups. The mood-related group was analyzed separately for prominent items, which were sadness, anxiety, pessimism, loss of interest and irritability. Wording of items were modified to be consistent with being collected throughout the day, rather than retrospectively. These items were incorporated as core observational domains in the application to be tested. Sadness and anxiety were additionally included as self-report items as studies have shown these to be most discordant between individuals with dementia and informants. Conclusions: This research is a first step towards an innovative approach to assessing depression in dementia. Next steps involve evaluation of the application’s feasibility and reliability for assessing depression in dementia.

